# Non-invasive imaging of high-risk coronary plaque: the role of computed tomography and positron emission tomography

**DOI:** 10.1259/bjr.20190740

**Published:** 2019-12-18

**Authors:** Rong Bing, Krithika Loganath, Philip Adamson, David Newby, Alastair Moss

**Affiliations:** 1 British Heart Foundation Centre for Cardiovascular Science, University of Edinburgh, Edinburgh, UK; 2 Wessex Heart Centre, University Hospital of Southampton, Southampton, UK; 3 Christchurch Heart Institute, University of Otago, Christchurch, New Zealand

## Abstract

Despite recent advances, cardiovascular disease remains the leading cause of death globally. As such, there is a need to optimise our current diagnostic and risk stratification pathways in order to better deliver individualised preventative therapies. Non-invasive imaging of coronary artery plaque can interrogate multiple aspects of coronary atherosclerotic disease, including plaque morphology, anatomy and flow. More recently, disease activity is being assessed to provide mechanistic insights into *in vivo* atherosclerosis biology. Molecular imaging using positron emission tomography is unique in this field, with the potential to identify specific biological processes using either bespoke or re-purposed radiotracers. This review provides an overview of non-invasive vulnerable plaque detection and molecular imaging of coronary atherosclerosis.

## Introduction

Despite recent improvements in the identification of coronary artery disease, fatal myocardial infarction remains the leading cause of death globally. In 2016, an estimated 17.6 million deaths were due to cardiovascular disease, of which 9.5 million were attributed to coronary artery disease.^1^ The increased use of evidence-based therapies combined with lifestyle modification has dramatically reduced complications from myocardial infarction over the past two decades. However, there remains an unmet clinical need to identify individuals with a high residual risk of recurrent cardiovascular events.^2^ Importantly, whilst validated risk scores can provide clinicians with information on expected survival following a myocardial infarction, they do not accurately predict which individuals will be at risk of recurrent plaque rupture events.^3^ To address this, research programmes are exploring whether novel imaging biomarkers can improve patient risk stratification to determine who may benefit from intensification of therapy.

Atherosclerotic plaque rupture resulting in luminal thrombosis and coronary artery occlusion is the most frequent cause of myocardial infarction. The vulnerable plaque model, which centres on identifying plaques with specific characteristics that are prone to rupture, has been a cornerstone of our understanding of myocardial infarction for decades.^4,5^ In recent years, there has been increasing recognition that most plaque rupture events remain clinically silent.^6^ Identifying vulnerable plaques alone may therefore be insufficient to provide incremental prognostic information above and beyond measures of total atherosclerotic plaque burden.^7^ Furthermore, there has been a shift in focus away from the assessment of luminal stenosis severity towards identification of high-risk plaque characteristics within the arterial wall, as the majority of *de novo* plaque rupture events occur in patients with non-obstructive stenoses.^7–9^ Non-invasive imaging offers a unique insight into disease processes that underpin atherosclerosis and may potentially identify novel therapeutic targets. In this review, we focus on the roles of CT coronary angiography (CTCA) and combined positron emission tomography and CT (PET-CT) in the assessment of vulnerable plaque and coronary artery disease activity.

## Pathophysiology of vulnerable plaque

Atherosclerosis is a chronic inflammatory disease that progresses over many years.^9^ Beginning relatively early in life, arterial endothelial damage leads to accumulation of vascular smooth muscle cells and intimal thickening. This process is more common in areas of low shear stress such as arterial bifurcations and is followed by intimal deposition of plasma lipoproteins, leading to oxidation and cellular apoptosis. The local inflammatory response is propagated by the expression of cellular adhesion molecules on vascular smooth muscle cells which promote migration and differentiation of circulating monocytes. Macrophage phagocytosis of lipid results in foam cells, which are the prelude to the lipid rich, necrotic core that is one of the hallmark features of vulnerable plaque. Other key histological and intravascular imaging characteristics of these lesions include macrophage infiltration, microcalcification, intraplaque haemorrhage and a thin fibrous cap.^10^ These vulnerable plaque features are targets that may be readily identified using a combination of anatomical and molecular imaging.

## Computed tomography coronary angiography to assess plaque vulnerability

CTCA offers both diagnostic and prognostic information for patients with coronary artery disease, with current scanners and protocols offering rapid prospective electrocardiography-gated imaging at low radiation doses. The ability to exclude obstructive coronary atherosclerosis is relevant for patients with suspected angina, and CTCA excels in this regard with a negative predictive value approaching 100% in some cohorts.^11^ The main impediments to accurate assessment of stenotic severity are (1) the presence of extensive coronary calcification or coronary stents which may cause photon starvation and partial volume artefact, thereby limiting accurate luminal assessment, and (2) inability to achieve adequate heart rate control, thereby increasing motion artefact. Two recent randomised controlled trials assessing the use of CTCA in stable patients have been published. The Scottish Computed Tomography of the Heart (SCOT-HEART) trial (*n* = 4146) compared CTCA to routine care in stable patients with suspected angina and demonstrated CTCA was associated with increased diagnostic certainty and more appropriate use of invasive angiography and preventative therapies.^12^ At 5 years, a pre-specified secondary analysis demonstrated a reduction in the combined endpoint of coronary heart disease death or non-fatal myocardial infarction in the CTCA arm [2.3% *vs* 3.9%, hazard ration (HR) 0.59, 95% confidence interval (CI) 0.41–0.84], principally driven by a reduction in non-fatal myocardial infarction (2.1% *vs* 3.5%, HR 0.60, 95% CI 0.41–0.87).^13^ To date, this is the only randomised controlled trial of non-invasive cardiac imaging that has demonstrated improved outcomes in this setting. Of note, approximately half of patients with subsequent adverse events did not have obstructive coronary artery disease. The Prospective Multicenter Imaging Study for Evaluation of Chest Pain (PROMISE) trial (*n*  =  10,003) compared CTCA with functional testing in symptomatic outpatients without diagnosed coronary artery disease in whom physicians believed non-urgent, non-invasive testing for suspected coronary artery disease was required.^14^ There was no difference in the primary outcome of death, myocardial infarction (MI), hospitalisation for unstable angina, or major procedural complication between groups (HR 1.04, 95% CI 0.83–1.29) at a mean follow-up of 25 months. However, the risk of death or non-fatal myocardial infarction was lower in the CTCA group at 12 months (HR 0.66, 95% CI 0.44 to 1.00).

On the basis of currently available data, the use of CTCA in patients with suspected stable angina is recommended as an appropriate first-line investigation in the 2019 European Society of Cardiology chronic coronary syndromes guidelines.^15^ Conversely, the role of CTCA in screening asymptomatic patients for primary prevention is not supported at present^16^ and given that CTCA requires the use of ionising radiation and iodinated contrast, this should not be undertaken routinely outside the context of a clinical trial. This question will be addressed in the upcoming CTCA for the Prevention of Myocardial Infarction (SCOT-HEART2) randomised controlled trial (NCT03920176).

**Figure 1.**
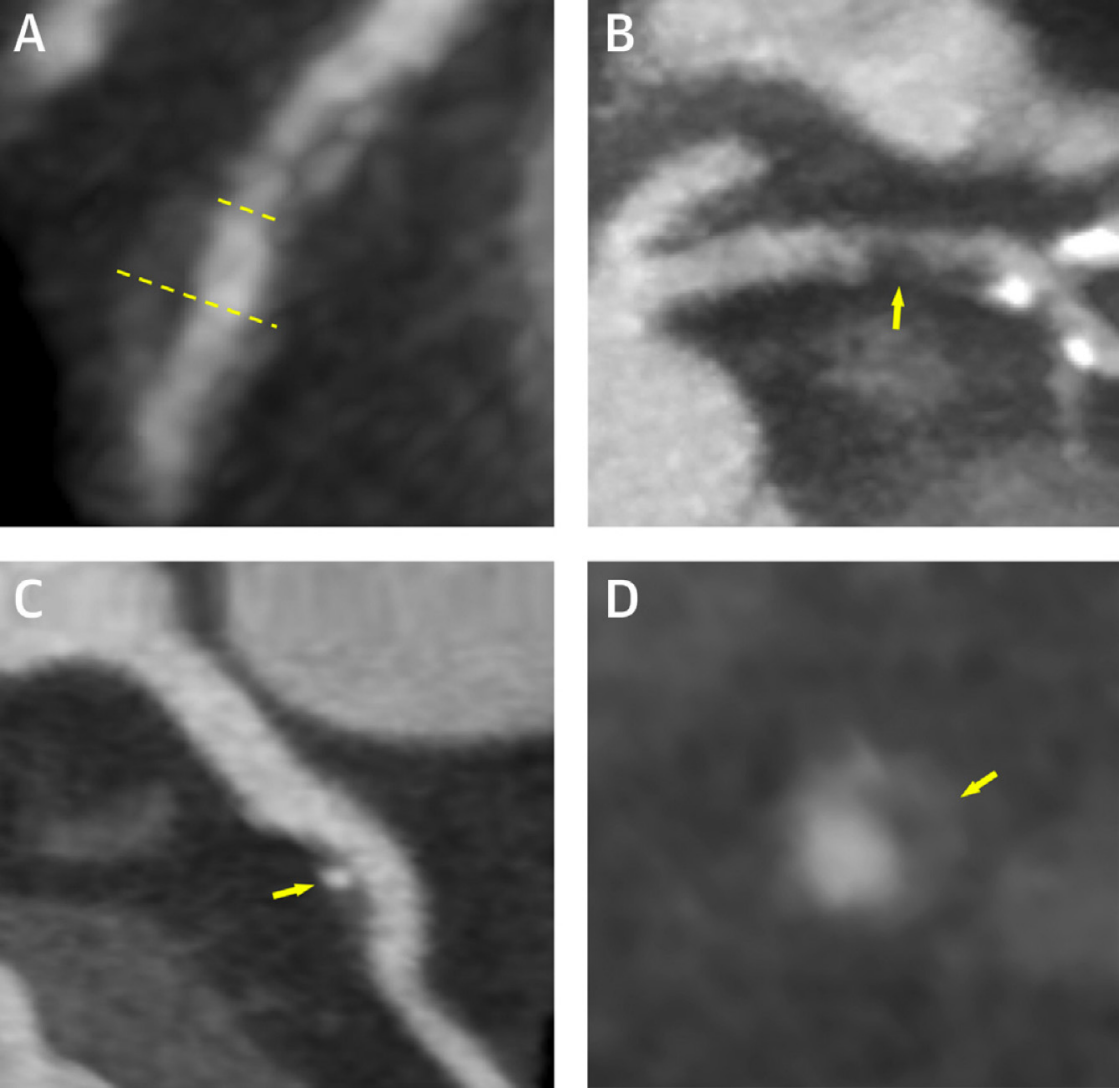
High-risk plaque features on CT coronary angiography. Features of high-risk atherosclerotic plaque including (A) positive remodelling, (B) low attenuation plaque, (C) spotty calcification and (D) the ‘napkin ring’ sign. Images courtesy of Williams et al.^7^

In addition to its diagnostic role in identifying obstructive coronary artery disease, CTCA is able to delineate intramural and extramural plaque characteristics, comparing favourably with intravascular ultrasound—long held to be the gold-standard for coronary atherosclerosis imaging.^17,18^ The hallmark CTCA findings of vulnerable plaque are low-attenuation plaque (<30 Hounsfield units), positive remodeling (maximum vessel diameter divided by reference vessel diameter >1.1), spotty calcification and the napkin-ring sign (low attenuation plaque core with a rim of high attenuation (Figure 1).^19^ Contemporary non-randomised data have consistently demonstrated the prognostic power of CTCA in identifying vulnerable plaque and predicting future acute coronary syndromes.^7,20–24^ These data are supported by analyses from SCOT-HEART and PROMISE. In SCOT-HEART, 608 (34%) patients had adverse plaque features which were associated with a 3-fold higher risk of coronary heart disease death or nonfatal myocardial infarction (HR 3.01, 95% CI 1.61–5.63).^7^ In PROMISE, 676 (15%) patients had high-risk plaque, the presence of which was again associated with a greater risk of major adverse events (HR 1.72, 95% CI 1.89–3.93).^25^ It is important to note that despite these associations, the high prevalence of high-risk plaque demonstrates the low positive predictive value of these findings for clinical events.

The clinical utility of identifying high-risk plaque remains unknown, as no additional intervention has been shown to reduce future cardiovascular risk above and beyond the use of optimal medical therapy in this setting. Recently, the Progression of Atherosclerotic Plaque Determined by Computed Tomographic Angiography Imaging (PARADIGM) study set out to evaluate the medium-term effects of statins on high-risk plaque formation and plaque progression in a low risk population.^26^ An eligible cohort of 1255 was divided into 474 statin-naive and 781 statin-taking patients. Of note, moderate or high-intensity statins (atorvastatin or rosuvastatin) were used in 94% of statin-taking patients. The rate of coronary artery disease progression was slower in patients receiving statin therapy compared with statin-naive patients (1.76 ± 2.4% vs. 2.04 ± 2.47% atheroma volume per year; *p* = 0.002) with no overall change in the rate of obstructive (>50% diameter) stenoses. Over a median follow-up of 3.4 years, statins increased the overall calcium volume, decreased the volume of non-calcified material and importantly reduced the rate of high-risk plaque development by 35%.^26^

## Coronary positron emission tomography to assess disease activity

The features of plaque vulnerability identified using CTCA may reflect structural changes in the content of the plaque that progress or regress over many years. Attention is now focused on developing non-invasive imaging techniques that may better assess short and medium-term cardiovascular risk. In this regard, PET-CT using targeted radiotracers to identify ligands involved in atherosclerotic pathophysiology has important clinical potential (Table 1). Radiotracers originally developed for imaging in oncology have been explored in the more challenging field of cardiovascular molecular imaging. Due to the small size of the coronary arteries and constant motion of the heart throughout the cardiac cycle, there has been significant technical investment in optimising acquisition protocols,^27–29^ reconstruction algorithms^30^ and post-processing interpretation of coronary artery activity to attain a robust methodology for molecular imaging in the coronary arteries.^31^ Here, we will focus on two radiotracers that have the most clinical potential; several other tracers have been investigated in small studies and are discussed elsewhere.^32^

**Table 1.** PET radiotracers under investigation to assess coronary plaque vulnerability

Target	Radiotracer	Study type	Summary of evidence to date	Selected references
Microcalcification	^18^F-Fluoride	*In vitro* experiments	Histological validation of selectivity for microcalcification	33, 34
		Technical feasibility studies	Good interobserver and scan-rescan repeatability	31
			Improvement in coronary assessment with: (1) Motion correction and blood pool clearance	21
			(2) 3 h delay injection – PET acquisition	29
			PET fusion with offline coronary CT angiography	44
			Optimised image reconstruction	30
			Partial volume correction for coronary arteries	45
		Prospective studies in stable CAD	Correlation with high risk plaque features	46
			Correlation with high risk phenotype	35, 47
			Measure of CAD activity in diabetes mellitus	48
			Intensification of antiplatelet therapy	38
		Prospective studies in acute coronary syndrome	Identifies culprit plaque rupture	37
Thrombus	^18^F-GP1	Phase I studies	High affinity in platelet aggregation	40
			First-in-human study carotid thrombosis	42

CAD, coronary artery disease; PET, positron emission tomography.

## 18F-fluoride

**Figure 2.**
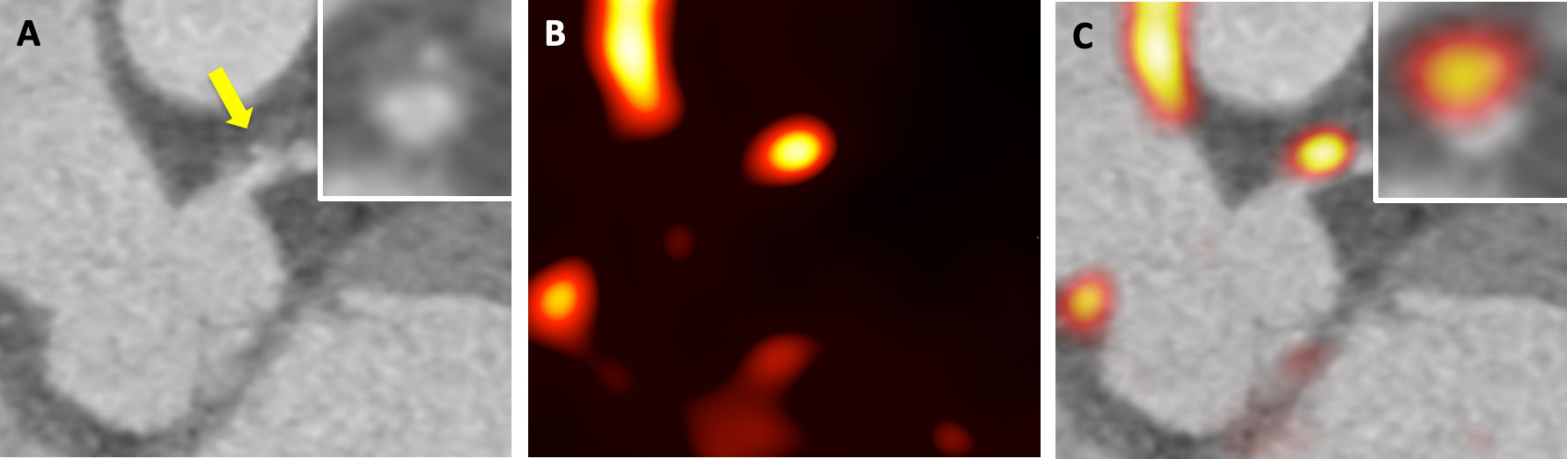
18F-fluoride uptake in left main stem high-risk coronary plaque. A 62-year-old male with anterior ST elevation myocardial infarction (high-sensitivity Troponin I 22144 ng l^−1^). (A) Following primary percutaneous coronary angiography, CT coronary angiography demonstrated a positively remodelled plaque with intraplaque haemorrhage and low attenutation in the distal left main stem (yellow arrow). *Inset* Cross-section multi planar reconstruction of distal left main stem lesion. (B & C) 18F-fluoride ECG-gated positron emission tomography revealed increased radiotracer uptake in the lesion. ECG, electrocardiogram.

**Figure 3.**
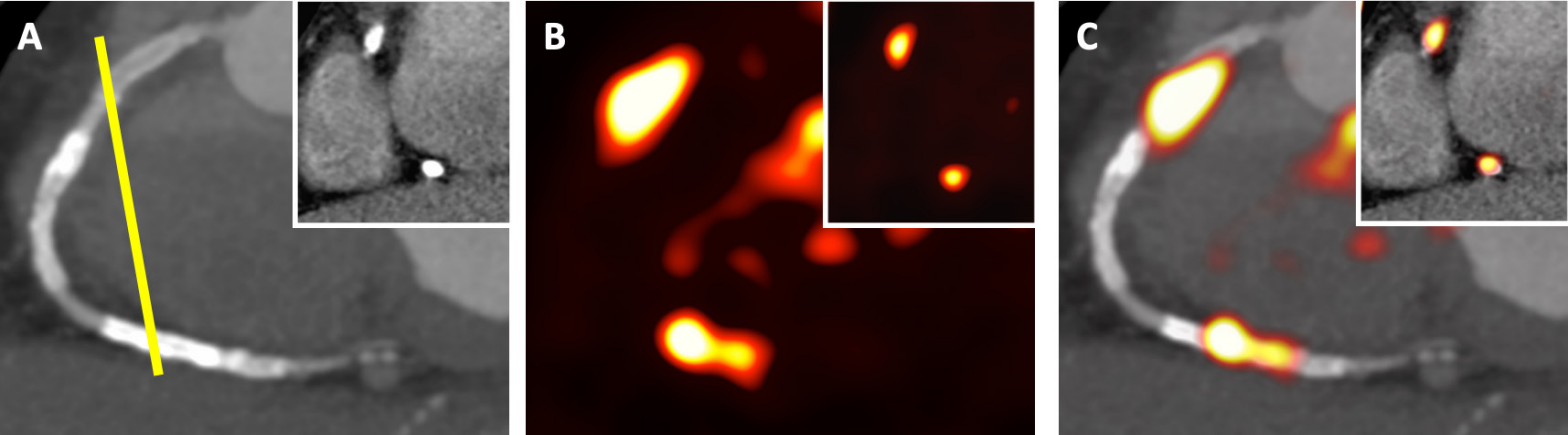
18F-fluoride uptake in right coronary artery. A 56-year-old male with inferior ST elevation myocardial infarction (high-sensitivity Troponin I 22910 ng l^−1^). (A) CT angiography following percutaneous coronary intervention to the proximal (culprit lesion) and mid-segment RCA. Previous coronary intervention was performed to distal RCA and PDA following ACS-NSTEMI 7 years prior to admission. *Inset* Sagittal reconstruction (yellow line) of proximal and distal RCA stents in the right atrioventricular groove. (B & C) 18F-fluoride ECG-gated positron emission tomography demonstrating accummulation of radiotracer in the proximal (culprit) and distal (old) RCA segments. CA, right coronary artery.

To date, 18F-sodium fluoride (18F-fluoride) is currently the most promising radiotracer for molecular coronary imaging due to its low background activity in the myocardium and excellent signal to noise ratio in all epicardial coronary arteries.^31^ The mechanism of 18F-fluoride binding in atherosclerotic coronary plaque is through hydroxyl exchange on the surface of hydroxyapatite to generate fluoroapatite which serves as a marker of microcalcification.^33,34^ As the calcium crystal size decreases, the surface area for 18F-fluoride binding increases, resulting in an intensified signal in regions of isolated microcalcification.^34^ This allows regions of microcalcification to be identified prior to the development of macrocalcific deposits which are recognised in advanced stable coronary artery disease.^35^ In contrast to the stability of this macrocalcification, atherosclerotic microcalcification arising from apoptotic macrophages alters the structural integrity of the fibrous cap and is thought to increase the propensity to plaque rupture.^36^ This manifests clinically as acute coronary syndromes where 18F-fluoride has been associated with a high proportion of culprit plaque ruptures^37^ (Figures 2 and 3). Ongoing clinical studies are investigating whether the identification of microcalcification using 18F-fluoride can predict individuals at risk of recurrent myocardial infarction (NCT02278211).

For a diagnostic imaging modality to have utility in wider clinical practice, it is must be able to guide risk stratification and treatment allocation to improve clinical outcomes. Phase II clinical trials using PET radiotracers have demonstrated good agreement between modification of plaque activity and subsequent larger Phase III clinical outcome studies (Table 2). Instead of continuing to adopt a ‘one size fits all’ approach within clinical trial design, the use of imaging biomarkers to quantify plaque activity may be an alternative strategy to identify those individuals who have the most to gain from intensification of treatment. This approach was applied in the recently published Dual Antiplatelet Therapy to Reduce Myocardial Injury (DIAMOND) study.^38^ In the context of advanced stable coronary artery disease, clinical trials have demonstrated that cardiovascular events can be reduced with the extended use of dual antiplatelet therapy, although there is no overall reduction in all-cause mortality as prolonged therapy comes at the expense of higher rates of major bleeding.^39^ The DIAMOND study was undertaken to test the hypothesis concerning whether 18F-fluoride PET imaging could appropriately select patients who may benefit from the extended use of P2Y12 inhibition. The study population comprised of 202 patients who underwent coronary 18F-fluoride PET imaging and were randomised 1:1 to ticagrelor, a potent P2Y12 inhibitor, or placebo to assess whether intensification of antiplatelet therapy would reduce subclinical myocardial injury in patients with high-risk plaque. A per-protocol group of 191 patients was used for the primary analysis and excluded patients without a troponin sample at 30 days and in whom compliance with the study medication (ticagrelor or placebo) was less than 80%. Whilst coronary 18F-fluoride activity was associated with higher plasma high-sensitivity cardiac troponin I concentrations (geometric mean 3.8 ± 2.9 *vs* 2.5 ± 2.6 ng l^−1^, *p* = 0.004), there was no reduction in subclinical myocardial injury at 30 days or 1 year.^38^ Whilst the primary end point did not reach statistical significance, this type of novel trial design illustrates some key lessons for future studies in this field. A sophisticated understanding of plaque pathophysiology will help to select the appropriate target for the right patient group. The underlying reason for this result may be due to the specificity of 18F-fluoride in identifying atherosclerotic microcalcification rather than intra coronary thrombosis *per se*. Radiotracers visualising plaque activity (18F-fluoride, 18F-fluoro-2-deoxyglucose) may be best used to stratify plaque-directed therapy (*e.g.* interleukin-1 β antagonists, proprotein convertase subtilisin/kexin Type 9 inhibitors). In contrast, to reduce the risk of thrombosis and platelet aggregation, radiotracers targeting the coagulation cascade may need to be considered.

**Table 2.** Use of positron emission tomography imaging to guide inflammatory response to treatment

	18F-FDG study (+)	18F-FDG study (-)
**Clinical outcome (+**)	Atorvastatin^49,50^ Pioglitazone^51^	
**Clinical outcome (-**)		Dalceptrapib^52^ 5-lipooxygenase inhibitor^53^ Losmapimod^54^ Oxidised low density lipoprotein antibody^55^

FDG, fludeoxyglucose.

Phase II clinicaltrials demonstrating cardiovascular 18F-FDG activity responseto therapy compared with subsequent Phase IIIclinical outcome trials. Study references in brackets. 18F-FDG, 18F-FDG.

## 18F-GP1

Thrombus formation plays an integral role in the pathophysiology of coronary atherosclerosis and plaque destabilisation. This is particularly relevant in the current era of high-sensitivity cardiac troponin assays. The identification of myocardial injury alone is often clinically insufficient, as there may be several differential diagnoses with distinct pathologies, only one of which may be plaque rupture. To date, there has been no clinically applicable non-invasive imaging technique that specifically targets thrombus. The advent of 18F-GP1 is therefore of major clinical interest.

Plaque rupture leads to exposure of the thrombogenic lipid core and recruitment of platelets to the site. The glycoprotein IIb/IIIa (GPIIb/IIIa) receptor is expressed in high density on activated platelets. It is a member of the integrin family of cell surface proteins and undergoes activation upon stimulation from a variety of thrombogenic factors. Upon activation, GPIIb/IIIa binds to fibrinogen which results in cross-linking and thrombus formation. GPIIb/IIIa has been a therapeutic target in cardiology for many years, with inhibitors (tirofiban, abciximab and eptifibatide) commonly used in high-risk acute coronary syndromes upstream of, or during, percutaneous coronary intervention. 18F-GP1 is a novel, small molecule fiban–class ligand that binds with high affinity to activated GPIIb/IIIa. Binding is not significantly affected by aspirin or heparin.^40^

**Figure 4.**
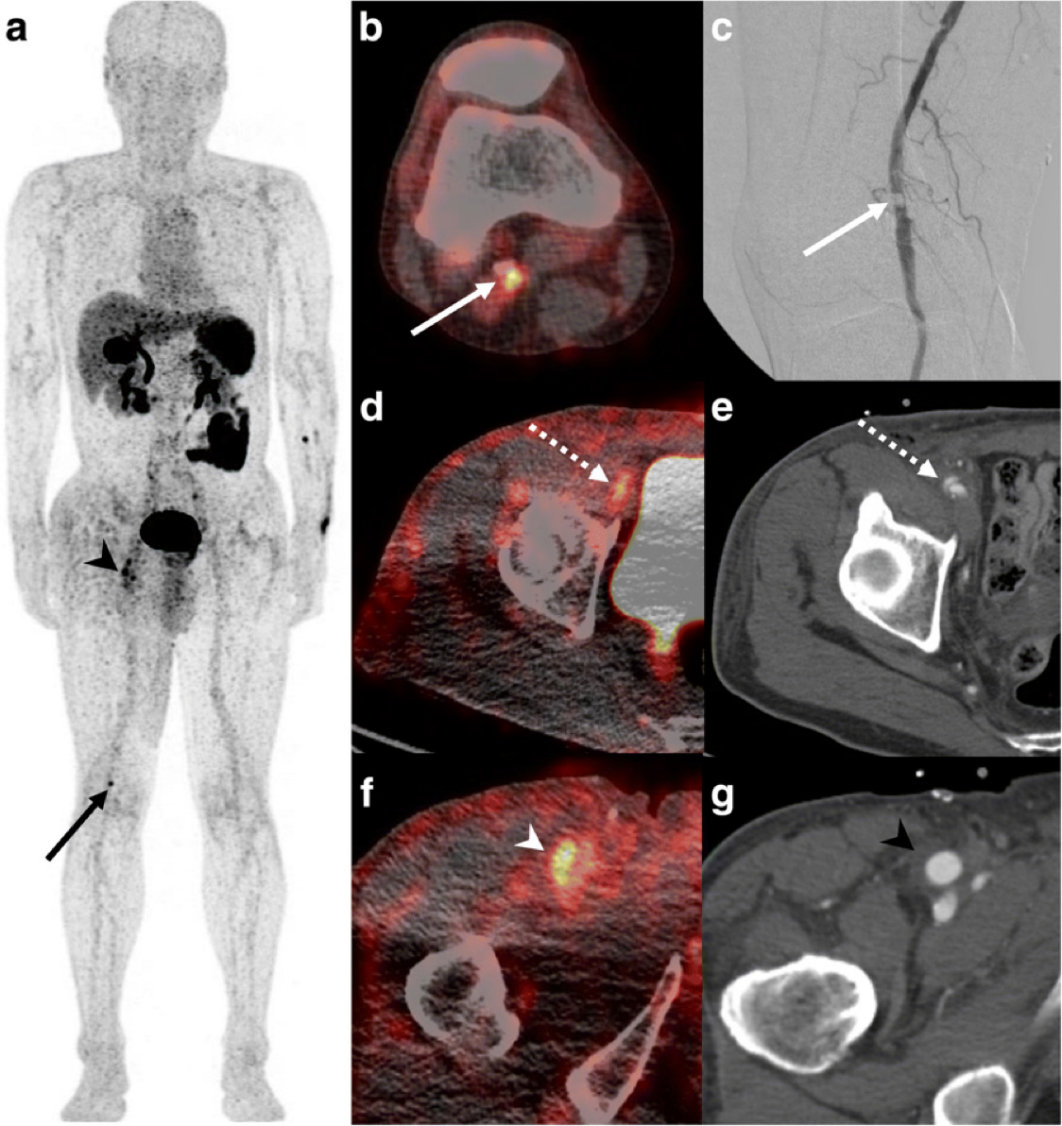
18F-GP1 arterial uptake in right popliteal artery. 18F-GP1 PET-CT images of a patient who had recently undergone right common femoral artery endarterectomy and right popliteal artery angioplasty. Anterior maximum intensity projection and axial images taken 120 min after 18F-GP1 injection show focal increased uptake in the right popliteal artery (a, b); arrows), which corresponds to a thrombotic lesion after angioplasty (c). Additional 18F-GP1 uptake is seen in the dissected right distal external iliac artery (d, e); dotted arrows) and right common femoral artery (a, f); arrow heads) where endarterectomy was performed 3 days prior to the PET-CT (g, arrow head). Images courtesy of Chae et al.^40^ PET, positron emission tomography.

Kim et al performed a first-in-human investigational study of 18F-GP1 in patients with acute deep vein thrombosis (*n* = 10) or pulmonary embolism (*n* = 10).^41^ The radiotracer was well-tolerated with initial high uptake followed by rapid clearance in the spleen, kidneys and blood pool. 18F-GP1 PET-CT detected 89% of vessels with deep vein thrombosis and 60% with pulmonary embolism, and interestingly demonstrated increased uptake in 32 vessels that were not detected by conventional imaging.^40^ The mean standardised uptake value of thromboemboli to blood pool ratio was 4.9 ± 1.4 and 3.7 ± 1.5 for deep vein thrombosis and pulmonary embolism respectively; a signal to background ratio that is superior to 18F-fluoride. Following this, the first-in-human study of 18F-GP1 in arterial thrombosis (six endovascular repair of abdominal aortic aneurysm, one bypass surgery and stent placement, one endarterectomy, one arterial dissection, and one acute cerebral infarction) was published by Chae et al.^42^ The investigators found uptake in arterial thrombus in all patients, again demonstrating a high mean standardised uptake value of thrombus to blood pool ratio (3.4, range 2.0–6.3) (Figure 4). There were no study-related adverse events. The favourable safety and radiation dosimetry profile of 18F-GP1 has been demonstrated and is comparable to other PET radiotracers.^43^ In light of these preliminary data, 18F-GP1 is a novel, highly promising radiotracer for coronary atherosclerosis imaging. The clinical applications of this specific thrombus tracer are broad-ranging, particularly for the identification of ruptured plaque and coronary thrombus. This has the potential to offer diagnostic information in patients with myocardial injury that cannot be acquired with any other currently available non-invasive imaging technique. The clinical use of 18F-GP1 requires combined PET-CT, thus offering anatomical and lesion-based assessments of plaque in addition to thrombus activity. Further clinical studies are highly anticipated.

## Conclusions

The emergence of hybrid non-invasive imaging modalities which can detect high-risk plaque offers new insights into the pathophysiology of coronary artery disease. Using non-invasive imaging to predict clinical outcomes and stratify therapy more appropriately are key objectives of current cardiovascular imaging research programmes. Whether the identification of these imaging phenotypes can improve the delivery of cardiovascular care will be addressed by ongoing clinical trials.
